# Systemic inflammatory indices and lung cancer risk by smoking and alcohol use in Korean men: A secondary dataset analysis of the Korean Cancer Prevention Study-II

**DOI:** 10.18332/tid/226556

**Published:** 2026-09-08

**Authors:** Jong Won Shin, Thien Minh Nguyen, Sun Ha Jee

**Affiliations:** 1 Department of Laboratory Medicine, Asan Medical Center, University of Ulsan College of Medicine, Seoul, Republic of Korea; 2 Department of Epidemiology and Health Promotion, Institute for Health Promotion, School of Public Health, Yonsei University, Seoul, Republic of Korea; 3 Department of Epidemiology, Faculty of Public Health, University of Medicine and Pharmacy, Ho Chi Minh City, Vietnam; 4 Department of Transdisciplinary Healthcare Sciences, Graduate School of Transdisciplinary Health Science, Yonsei University, Seoul, Republic of Korea

**Keywords:** lung neoplasms, systemic immune-inflammation index, neutrophil-to-lymphocyte ratio, inflammation, smoking

## Abstract

**INTRODUCTION:**

Systemic inflammation has been implicated in cancer development, but prospective evidence regarding its association with lung cancer risk remains limited. This study evaluated the associations of systemic immune-inflammation index (SII) and neutrophil-to-lymphocyte ratio (NLR) with lung cancer risk according to smoking status, alcohol drinking status, and their combined exposure patterns in Korean men.

**METHODS:**

This secondary dataset analysis utilized data from the Korean Cancer Prevention Study-II (KCPS-II), a nationwide prospective cohort. Among 20167 men, 236 incident lung cancer cases were identified during a median follow-up of approximately 14 years. Adjusted hazard ratios (AHRs) for lung cancer per 1 standard deviation (SD) increase in SII and NLR were estimated using multivariable Cox proportional hazards models adjusted for demographic, lifestyle, liver function, and endogenous antioxidant biomarkers. Analyses were performed in the overall population and stratified by smoking and alcohol status, including concurrent current smokers and drinkers.

**RESULTS:**

Higher SII and NLR were associated with increased lung cancer risk in Korean men, with consistent associations observed among current smokers, current drinkers, and individuals with concurrent current smoking and drinking. In combined exposure analyses, substantially elevated risks were observed among current smokers with high inflammatory marker levels (SII: AHR=3.58; 95% CI: 2.51–5.09; NLR: AHR=3.78; 95% CI: 2.65–5.39). SII showed the strongest association among individuals smoking ≥30 cigarettes/day (AHR=1.39; 95% CI: 1.21–1.60).

**CONCLUSIONS:**

Systemic inflammatory markers, particularly SII, were associated with lung cancer risk in Korean men, with stronger associations observed among current smokers. These findings suggest that systemic inflammatory status may help identify individuals at elevated risk of smoking-related lung cancer.

## INTRODUCTION

Lung cancer remains the leading cause of cancer-related mortality worldwide^1^, and cigarette smoking is well established as the most important risk factor for its development^2,3^. However, substantial inter-individual variation in lung cancer risk exists even among individuals with similar levels of smoking exposure^3^, and alcohol consumption has also been suggested as a lifestyle factor associated with lung cancer risk^4-6^. This heterogeneity in risk suggests that, beyond behavioral exposures such as smoking and alcohol consumption, underlying biological states, including systemic inflammation, may contribute to variation in lung cancer susceptibility. In particular, systemic inflammation and oxidative stress are key processes involved in cancer initiation and progression^7,8^. Systemic inflammatory markers, including the systemic immune-inflammation index (SII) and the neutrophil-to-lymphocyte ratio (NLR), have been widely used as indicators of inflammatory and immune status^9-12^. NLR reflects the balance between innate and adaptive immune responses, whereas SII integrates platelet, neutrophil, and lymphocyte components to provide a more comprehensive measure of systemic inflammation and immune regulation^13,14^. Recent studies have reported that these markers are associated with prognosis and disease progression across various cancers^15-19^, and some studies have also suggested their associations with cancer incidence^20^. However, most previous studies have focused on patient populations or have been based on cross-sectional or limited study designs, and prospective evidence evaluating their associations with lung cancer risk in general populations remains limited^21,22^. Lifestyle factors such as smoking and alcohol consumption are closely linked to systemic inflammatory status and may influence lung carcinogenesis through complex and interrelated pathways rather than as isolated exposures^23,24^. Nevertheless, prior studies have largely evaluated inflammatory markers and lifestyle factors independently, and prospective studies examining their combined effects remain scarce^25^. In addition, few studies have incorporated quantitative exposure measures, such as smoking intensity and alcohol consumption levels, when assessing the relationship between systemic inflammation and lung cancer risk^21^. Therefore, we conducted a secondary analysis of data from the Korean Cancer Prevention Study-II (KCPS-II), an established prospective cohort of Korean men, to evaluate the associations of SII and NLR with lung cancer risk and to examine whether these associations vary according to smoking and alcohol status. We further assessed associations according to smoking intensity and alcohol consumption levels and investigated combined exposure patterns of systemic inflammatory markers and behavioral risk factors in relation to lung cancer risk.

## METHODS

### Study population

**Figure 1:**
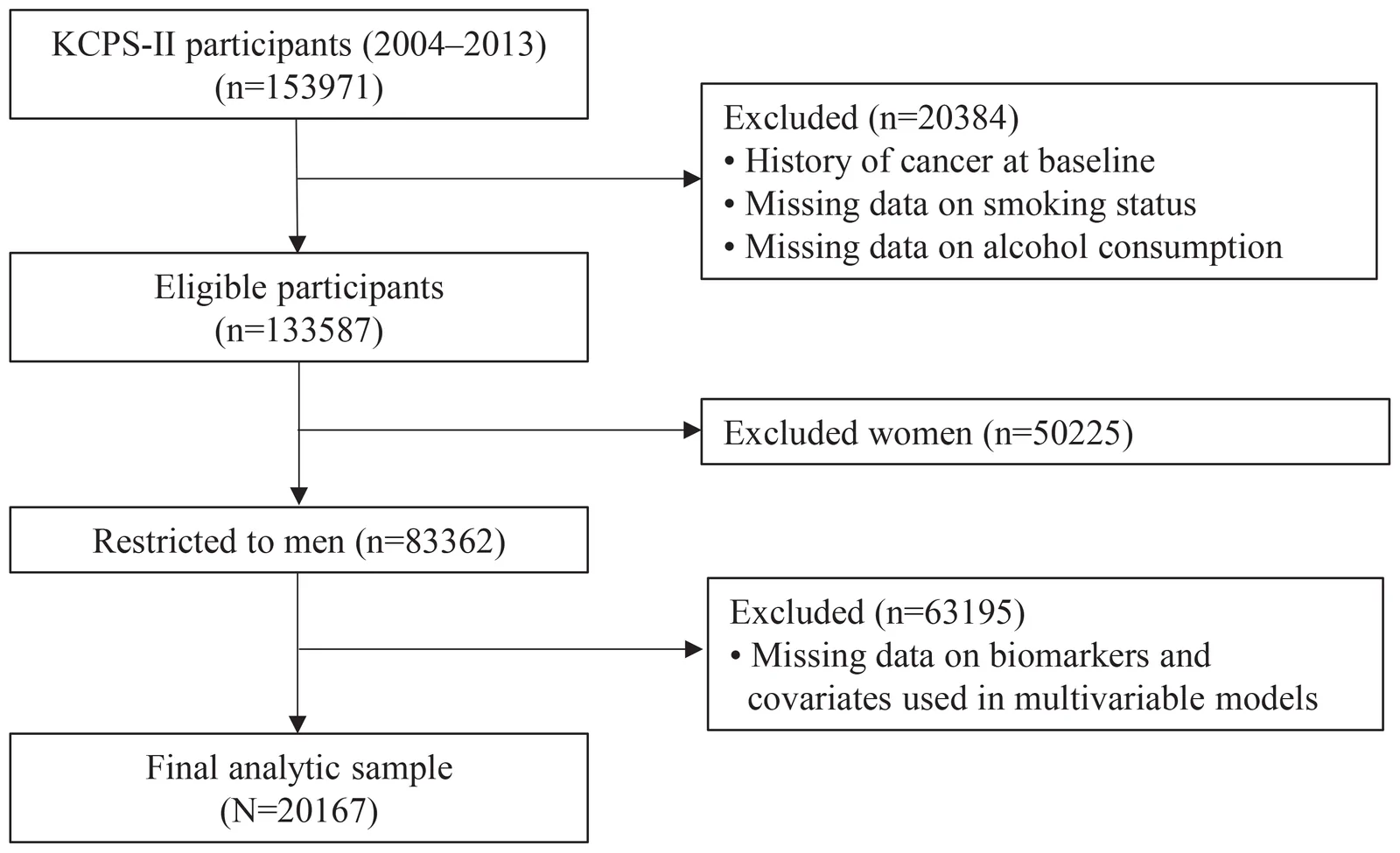
Flow diagram of study participants

This secondary dataset analysis utilized data from the Korean Cancer Prevention Study-II (KCPS-II), a nationwide prospective cohort comprising 153971 adults aged ≥20 years who visited 18 health screening centers across Korea between 2004 and 2013. All participants provided written informed consent for the use of their health screening and questionnaire data for research purposes^26,27^. Individuals with a history of cancer at baseline were excluded. For the present analysis, the study population was restricted to men, given the very low prevalence of smoking among women in this cohort, which limits the statistical power to evaluate smoking- and alcohol-related associations in women. Participants with missing information on smoking or alcohol drinking status were excluded. In addition, individuals with missing values for neutrophil, lymphocyte, or platelet measurements required to calculate systemic inflammatory indices were excluded. Furthermore, to ensure consistency in multivariable analyses, participants with missing measurements of liver function markers (GOT and GGT) or endogenous antioxidant biomarkers, including bilirubin, albumin, and uric acid, were excluded. The final analytical sample consisted of 20167 men (Figure 1). This study was conducted in accordance with the Declaration of Helsinki and was approved by the Institutional Review Board of Severance Hospital (approval number: 4-2011-0277).

### Cancer case ascertainment

Cancer incidence among study participants was ascertained through annual linkage to the National Cancer Center (NCC) Registry using unique resident registration numbers. The NCC Registry provides near-complete coverage of cancer diagnoses in Korea, as reporting of all cancer cases from medical institutions is mandated under the Cancer Control Act, ensuring high completeness and validity of cancer ascertainment^26^. Cancer cases were classified according to the International Classification of Diseases, 10th Revision (ICD-10), and lung cancer (C34) was defined as the primary outcome in this study. During the follow-up period, a total of 236 incident lung cancer cases were identified among men. Of these, 126 cases occurred among current smokers, 194 among current drinkers, and 102 among individuals who were both current smokers and drinkers. The total follow-up person-years was 285563.55 and the incidence rate of lung cancer was 82.6 per 100000 person-years. The median duration of follow-up was 14.0 years (interquartile range, IQR: 13.3–14.5).

### Statistical analysis

Baseline characteristics of the study population were summarized using descriptive statistics. Continuous variables were presented as mean and standard deviation (SD), and categorical variables were presented as frequencies and percentages. Missing data were handled using a complete-case approach. Participants with missing information on covariates or biomarker measurements required for the analyses, were excluded. The potential impact of missing data was considered when interpreting the findings.

The systemic immune-inflammation index (SII) and neutrophil-to-lymphocyte ratio (NLR) were standardized per 1 standard deviation (SD) increase. SII was calculated as platelet count × (neutrophil percentage/lymphocyte percentage), and NLR was calculated as the ratio of neutrophil percentage to lymphocyte percentage based on differential leukocyte data obtained from baseline complete blood cell measurements.

Cox proportional hazards regression models were used to evaluate the associations between SII and NLR and the risk of lung cancer. Multivariable models were constructed sequentially to evaluate the incremental effects of potential confounding factors. Model 1 was adjusted for age, body mass index, and family history of cancer, with additional adjustment for smoking status or alcohol drinking status depending on the stratified analysis. Model 2 was further adjusted for liver function markers, including glutamate oxaloacetate transaminase (GOT) and gamma-glutamyl transferase (GGT). Model 3 was additionally adjusted for endogenous antioxidant biomarkers, including bilirubin, albumin, and uric acid. The proportional hazards assumption was assessed using Schoenfeld residuals, and no meaningful violations were observed. Statistical significance was defined as a two-sided p<0.05.

SII and NLR were categorized into quartiles to evaluate their associations with lung cancer risk, with the lowest quartile (Q1) used as the reference group. Tests for trend were performed by modeling the median value of each quartile as a continuous variable. Stratified analyses were conducted according to smoking and alcohol drinking status, with participants categorized into all men, current smokers, current drinkers, and men with concurrent current smoking and drinking. In these stratified analyses, the stratifying variable was not additionally included in the model, while the other covariates were retained.

To assess combined exposure patterns of systemic inflammatory markers and behavioral risk factors, SII and NLR were categorized into quartiles, with the highest quartile (Q4) defined as high levels and the lower three quartiles (Q1–Q3) as low levels. Combined exposure groups were defined by combining inflammatory marker levels with current smoking or drinking status. In the smoking model, the reference group consisted of individuals with low inflammatory marker levels and non-current smoking, whereas in the alcohol model, the reference group consisted of individuals with low inflammatory marker levels and non-current drinking. Additional analyses according to smoking intensity and alcohol consumption levels were performed using 1-SD increases in SII and NLR.

Person-years of follow-up were calculated from the date of the baseline examination to the date of lung cancer diagnosis, death, or the end of follow-up, whichever occurred first. The original KCPS-II cohort was established to investigate associations between health-related factors and chronic disease outcomes, and participants have been followed through linkage with national cancer registration and mortality databases. All biochemical measurements were performed at certified laboratories adhering to internal and external quality control protocols established by the Korean Association of Laboratory Quality Control. Inter-laboratory correlation coefficients ranged from 0.96 to 0.99, indicating high accuracy and consistency.

All statistical analyses were conducted using SAS software (version 9.4; SAS Institute Inc., Cary, NC, USA), and figures were generated using R software (version 4.3.2; R Foundation for Statistical Computing, Vienna, Austria)^28,29^.

## RESULTS

### Characteristics of the study population

Baseline characteristics of the study population are presented in Table 1. A total of 20167 men were included in the analysis, with a mean age of 46.63 years (SD=10.12) and a mean body mass index of 24.63 kg/m^2^ (SD=2.82). The mean platelet count was 240.24 (SD=53.31), and the mean neutrophil and lymphocyte percentages were 54.00% (SD=8.39) and 35.29% (SD=7.49), respectively. The mean levels of total bilirubin, albumin, and uric acid were 1.03 mg/dL, 4.70 g/dL, and 5.93 mg/dL, respectively. Regarding smoking status, 21.07% were never smokers, 38.18% were former smokers, and 40.75% were current smokers. For alcohol consumption, 9.48% were abstainers, 3.27% were former drinkers, and 87.25% were current drinkers. A family history of cancer was reported in 22.39% of participants.

**Table 1:** Baseline demographic, clinical, lifestyle, and inflammatory characteristics of Korean men included in the Korean Cancer Prevention Study-II cohort (N=20167; baseline recruitment 2004–2013)

*Characteristics*	*Mean (SD)*
**Age** (years)	46.63 (10.12)
**Clinical status**	
Body mass index (kg/m^2^)	24.63 (2.82)
Platelet (10³/µL)	240.24 (53.31)
Neutrophil (%)	54.00 (8.39)
Lymphocyte (%)	35.29 (7.49)
Serum total bilirubin (mg/dL)	1.03 (0.43)
Albumin (g/dL)	4.70 (0.27)
Uric acid (mg/dL)	5.93 (1.26)
**Smoking status**, %	
Never smokers	21.07
Former smokers	38.18
Current smokers	40.75
**Alcohol drinking status**, %	
Abstainer	9.48
Former drinker	3.27
Current drinker	87.25
**Family history of cancer**, n (%)	4516 (22.39)

SII: systemic immune-inflammation index. NLR: neutrophil-to-lymphocyte ratio. Participants included in this table were restricted to men with available data on SII, NLR, smoking status, alcohol drinking status, and covariates used in the analysis.

The associations of SII and NLR with lung cancer risk per 1-SD increase are presented in Table 2. In the fully adjusted Model 3, among all men, SII was associated with an adjusted hazard ratio (AHR) of 1.13 (95% CI: 1.03–1.24; p=0.0083), and NLR was associated with an AHR of 1.12 (95% CI: 1.01–1.23; p=0.0280). Among current smokers, the AHR for SII was 1.15 (95% CI: 1.03–1.29; p=0.0134), whereas the association for NLR was not statistically significant (AHR=1.13; 95% CI: 0.98–1.32; p=0.1007). Among current drinkers, SII (AHR=1.17; 95% CI: 1.06–1.28; p=0.0010) and NLR (AHR=1.15; 95% CI: 1.04–1.27; p=0.0078) were significantly associated with lung cancer risk. Among individuals with concurrent current smoking and drinking, SII (AHR=1.18; 95% CI: 1.06–1.32; p=0.0037) and NLR (AHR=1.20; 95% CI: 1.01–1.41; p=0.0357) showed significant positive associations.

**Table 2:** Association of SII and NLR with lung cancer risk according to smoking and alcohol status in men

*Status*	*Cases*	*Person-years (py)*	*Incidence per 100000 py*	*SII*		*NLR*	
				*AHR (95% CI)*	*p*	*AHR (95% CI)*	*p*
**Model 1**							
All men	236	285563.55	82.6	1.15 (1.05–1.26)	0.0028	1.13 (1.03–1.25)	0.0144
Current smokers	126	116363.87	108.3	1.17 (1.05–1.31)	0.0061	1.15 (0.99–1.34)	0.0594
Current drinkers	194	249684.88	77.7	1.18 (1.08–1.30)	<0.001	1.16 (1.05–1.28)	0.0037
Current smokers and drinkers	102	106113.23	96.1	1.20 (1.08–1.35)	0.0013	1.21 (1.03–1.43)	0.0217
**Model 2**							
All men	236	285563.55	82.6	1.14 (1.04–1.25)	0.0039	1.13 (1.02–1.24)	0.0183
Current smokers	126	116363.87	108.3	1.16 (1.04–1.30)	0.0085	1.15 (0.99–1.33)	0.0726
Current drinkers	194	249684.88	77.7	1.18 (1.08–1.29)	<0.001	1.16 (1.05–1.28)	0.0048
Current smokers and drinkers	102	106113.23	96.1	1.20 (1.07–1.34)	0.0020	1.21 (1.02–1.43)	0.0255
**Model 3**							
All men	236	285563.55	82.6	1.13 (1.03–1.24)	0.0083	1.12 (1.01–1.23)	0.0280
Current smokers	126	116363.87	108.3	1.15 (1.03–1.29)	0.0134	1.13 (0.98–1.32)	0.1007
Current drinkers	194	249684.88	77.7	1.17 (1.06–1.28)	0.0010	1.15 (1.04–1.27)	0.0078
Current smokers and drinkers	102	106113.23	96.1	1.18 (1.06–1.32)	0.0037	1.20 (1.01–1.41)	0.0357

SII: systemic immune-inflammation index. NLR: neutrophil-to-lymphocyte ratio. AHR: adjusted hazard ratio; estimated per 1 standard deviation increase in SII and NLR. Model 1 was adjusted for age, family history of cancer, and body mass index. Smoking and alcohol variables were adjusted depending on the stratified group: alcohol variables were included in smoking-stratified analyses, smoking variables were included in alcohol-stratified analyses, and neither was included in analyses restricted to individuals with concurrent smoking and alcohol consumption. Model 2 was additionally adjusted for liver function markers (GOT and GGT). Model 3 was additionally adjusted for endogenous antioxidant biomarkers, including bilirubin, albumin, and uric acid.

**Figure 2:**
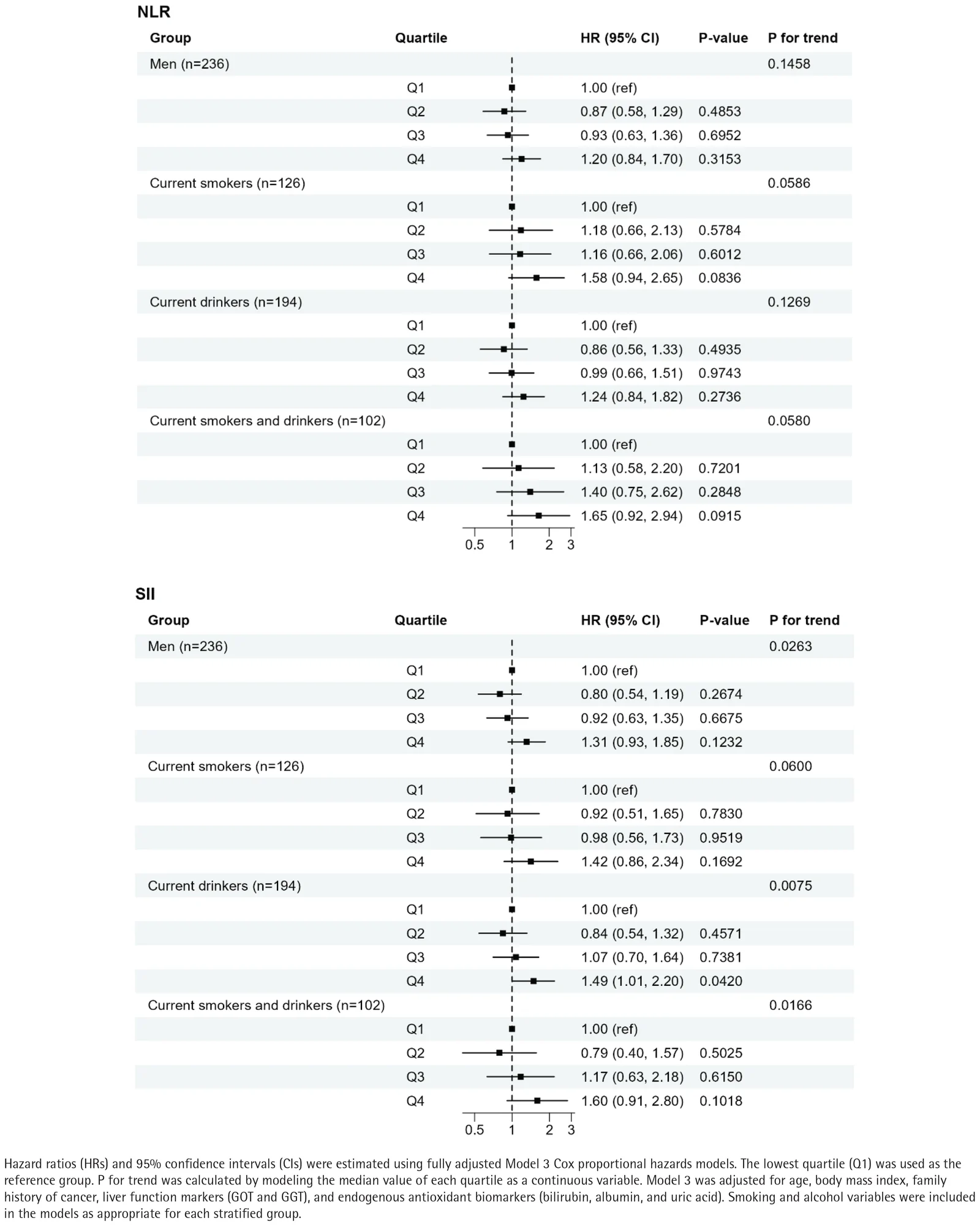
Associations of systemic inflammatory indices (SII and NLR) with lung cancer risk by quartiles, stratified by smoking and alcohol status in men

The associations of SII and NLR with lung cancer risk according to quartile categories are presented in Figure 2. Hazard ratios were estimated using fully adjusted Model 3 Cox proportional hazards models, with the lowest quartile (Q1) as the reference group. Among current drinkers, the highest quartile (Q4) of SII was associated with an increased risk of lung cancer (AHR=1.49; 95% CI: 1.01–2.20; p=0.0420), and a significant trend was observed for SII in the trend analysis.

**Figure 3:**
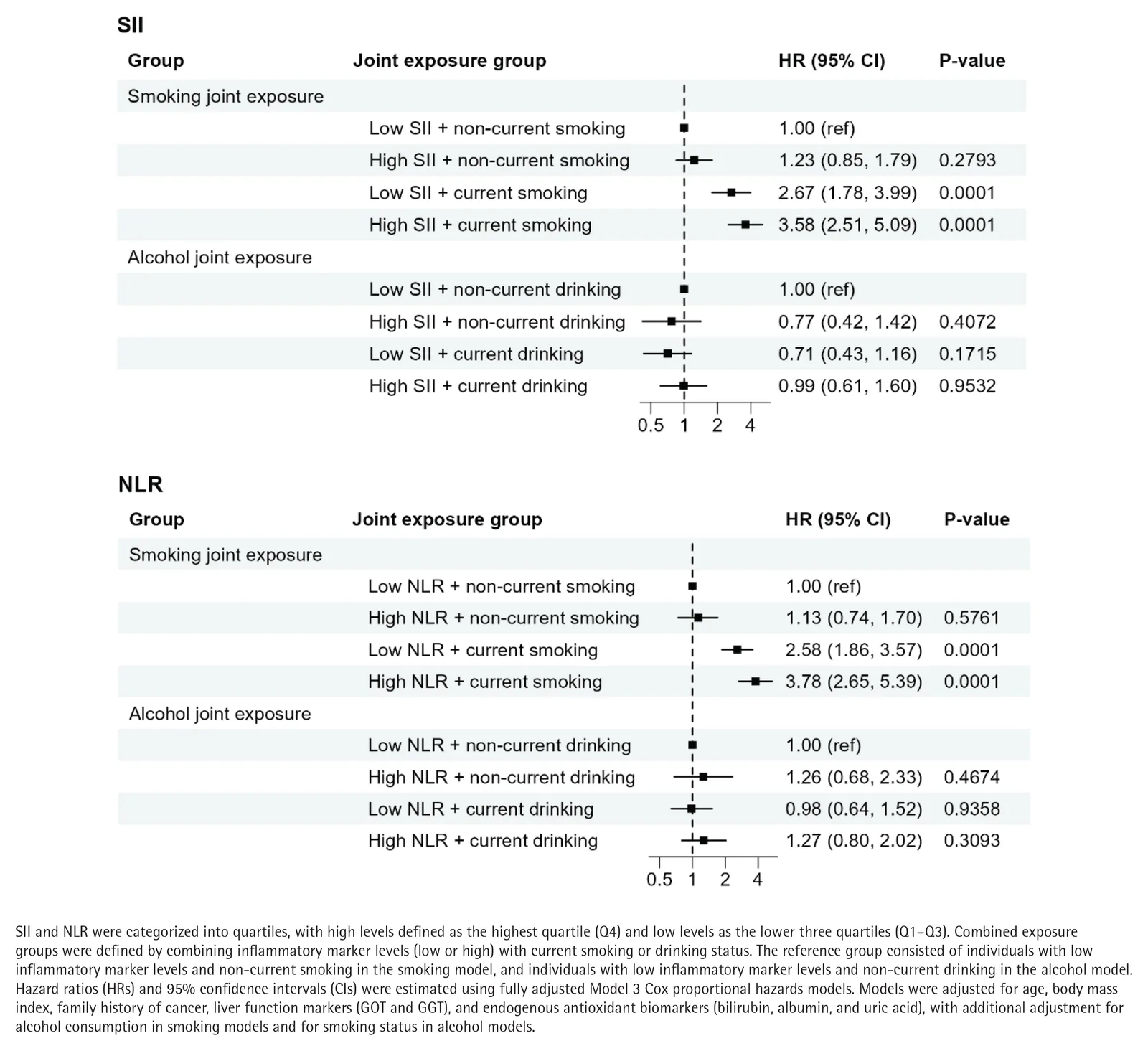
Associations of systemic inflammatory indices (SII and NLR) with lung cancer risk according to combined exposure patterns of smoking and alcohol use in men

The combined exposure patterns of systemic inflammatory markers with smoking and alcohol consumption are presented in Figure 3. SII and NLR were categorized into high (Q4) and low (Q1–Q3) levels, and combined exposure groups were defined by combining inflammatory marker levels with current smoking or drinking status.

Fully adjusted Model 3 Cox proportional hazards models were used. In the smoking model, the reference group consisted of individuals with low inflammatory marker levels and non-current smoking, whereas in the alcohol model, the reference group consisted of individuals with low inflammatory marker levels and non-current drinking.

Among individuals with current smoking, the AHR was 2.67 (95% CI: 1.78–3.99) for low SII and 3.58 (95% CI: 2.51–5.09) for high SII, while the corresponding AHRs for NLR were 2.58 (95% CI: 1.86–3.57) and 3.78 (95% CI: 2.65–5.39), respectively. Notably, even among individuals with low inflammatory marker levels, current smoking was associated with increased lung cancer risk, although the magnitude of association was greater among those with high inflammatory marker levels.

**Figure 4:**
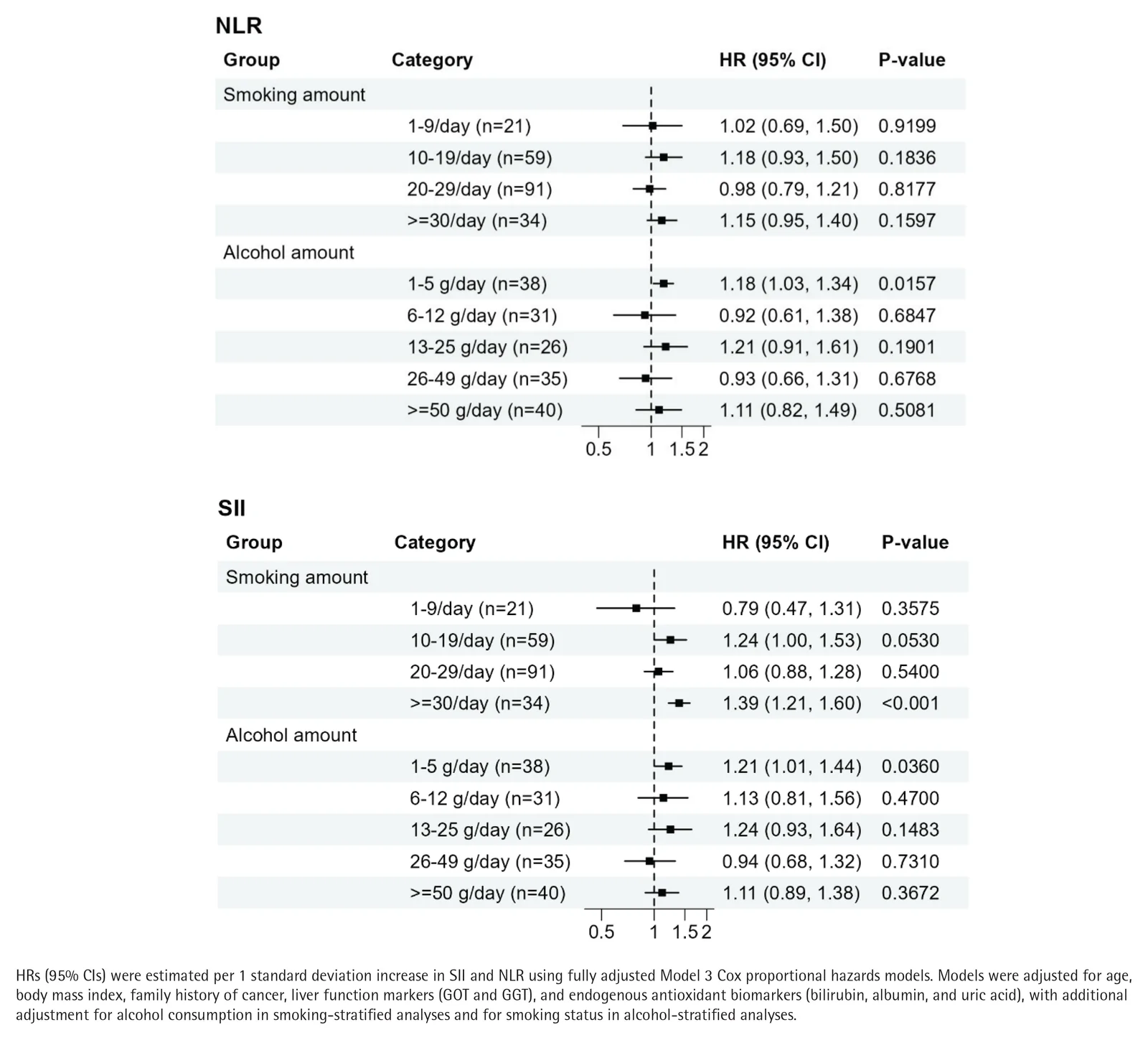
Associations of systemic inflammatory indices (SII and NLR) with lung cancer risk by smoking and alcohol amount in men

The associations of SII and NLR with lung cancer risk according to smoking intensity and alcohol consumption levels are presented in Figure 4. Hazard ratios were estimated per 1-SD increase in SII and NLR using fully adjusted Model 3 Cox proportional hazards models.

Among individuals who smoked ≥30 cigarettes per day, SII showed a significant association with lung cancer risk (AHR=1.39; 95% CI: 1.21–1.60). Among those with alcohol consumption of 1–5 g/day, SII (AHR=1.21; 95% CI: 1.01–1.44) and NLR (AHR=1.18; 95% CI: 1.03–1.34) were significantly associated with lung cancer risk.

## DISCUSSION

This study evaluated the associations between systemic inflammatory markers, namely the systemic immune-inflammation index (SII) and neutrophil-to-lymphocyte ratio (NLR), and lung cancer risk in a large prospective cohort of Korean men with approximately 14 years of follow-up. The main findings of this study are as follows. First, a one standard deviation increase in SII and NLR was positively associated with lung cancer risk in the overall male population, and these associations were also observed among current smokers, current drinkers, and individuals with concurrent current smoking and drinking. Notably, SII remained significantly associated with lung cancer risk across all groups even after additional adjustment for liver function markers and endogenous antioxidant biomarkers. Second, in the quartile analysis, the highest quartile of SII was significantly associated with lung cancer risk among current drinkers, and a significant trend was observed for SII. Third, in the joint exposure analysis, markedly elevated risks of lung cancer were observed in groups combining high SII or high NLR with current smoking (Figure 3). Fourth, in analyses stratified by smoking and alcohol consumption levels, a significant association for SII was observed among individuals smoking at least 30 cigarettes per day, while both SII and NLR showed significant associations in those consuming 1–5 g of alcohol per day. Overall, these findings suggest that systemic inflammatory markers may be associated with lung cancer risk, and that these associations may be more pronounced under conditions involving smoking.

The finding that SII showed more consistent results than NLR is noteworthy. SII incorporates neutrophils, lymphocytes, and platelets, and may therefore more comprehensively reflect systemic inflammatory and immune status compared to NLR. Increased neutrophil levels have been associated with the release of inflammatory mediators, reactive oxygen species, and proteolytic enzymes within the tumor microenvironment, while decreased lymphocyte levels may reflect impaired anti-tumor immune surveillance^30,31^. In addition, platelets are known to contribute to tumor cell survival, adhesion, and micrometastasis^32,33^, suggesting that SII may capture multiple biological pathways related to lung cancer development. These findings are consistent with the relatively stable associations observed for SII across Models 1–3 in this study. In contrast, although NLR showed significant associations in the overall population, current drinkers, and individuals with concurrent current smoking and drinking, its association was attenuated in the analysis restricted to current smokers. This may indicate that while NLR reflects certain aspects of smoking-related chronic inflammation, it may not provide as comprehensive information as SII.

Previous epidemiological studies have suggested that systemic inflammatory markers, including NLR and other inflammation-related indices, are associated with lung cancer development and prognosis. Large prospective studies have reported positive associations between circulating inflammatory biomarkers and subsequent lung cancer risk, supporting the potential role of chronic systemic inflammation in lung carcinogenesis^34^. However, most previous studies have evaluated inflammatory markers independently and have rarely incorporated detailed lifestyle-related inflammatory exposures, such as smoking intensity and alcohol consumption levels. Furthermore, many previous studies have focused on patient populations or clinical outcomes rather than incident lung cancer development in initially healthy populations. The present study extends previous evidence by evaluating systemic inflammatory markers in relation to incident lung cancer risk within a large population-based prospective cohort and by examining whether these associations differ according to smoking and alcohol exposure patterns. In particular, the stronger associations observed among current smokers suggest that systemic inflammatory markers may reflect the cumulative biological effects of smoking-related oxidative stress and immune dysregulation.

An important finding of this study is that the association of systemic inflammatory markers with lung cancer risk appeared stronger among men with current smoking, particularly in the joint exposure analysis. The markedly increased risk of lung cancer observed in groups combining high SII or high NLR with current smoking suggests that not only the harmful effects of smoking itself, but also the systemic inflammatory state accompanying smoking, may help identify high-risk populations (Figure 3). Smoking is a well-established risk factor for lung cancer that induces oxidative stress and chronic inflammatory responses and is associated with airway epithelial damage, infiltration of inflammatory cells, DNA damage, and dysregulation of immune responses^2,3^. In this context, elevated inflammatory markers may not merely represent a concurrent phenomenon but may serve as indicators of the intensity of the smoking-related carcinogenic environment. In particular, both SII and NLR were significant in the group with concurrent current smoking and drinking in the one standard deviation analysis, and the effect sizes for high SII and high NLR combined with current smoking were substantial in the joint exposure analysis. Taken together, the main message of this study is more appropriately centered on the combination of smoking and systemic inflammation rather than alcohol. In other words, lung cancer risk may be better characterized not only by smoking status alone but also by the underlying inflammatory biological state associated with smoking.

In contrast, the findings related to alcohol consumption should be interpreted with greater caution compared with smoking. Although significant associations were observed for SII and NLR in the one standard deviation analysis among current drinkers, and for the highest quartile of SII in the quartile analysis, the patterns observed in the joint exposure analysis were less pronounced than those involving smoking. In addition, consistent associations were not observed at the highest levels of alcohol consumption. These findings suggest that alcohol may have a weaker or less direct relationship with lung cancer risk compared with smoking, and that the measurement of alcohol consumption may not have fully captured long-term drinking behaviors. Furthermore, given the strong correlation between alcohol consumption and smoking, some of the observed associations among current drinkers may reflect overlapping lifestyle factors or general health behaviors rather than independent effects of alcohol. Therefore, while alcohol may contribute to the relationship between systemic inflammation and lung cancer risk to some extent, its effects appear less consistent and weaker than those of smoking.

The analyses according to smoking and alcohol consumption levels were largely consistent with these interpretations. The significant association observed for SII among individuals smoking at least 30 cigarettes per day suggests that systemic inflammatory status may better reflect lung cancer risk when combined with higher smoking intensity. This finding is consistent with the possibility that increased smoking intensity leads to greater cumulative oxidative stress and inflammatory stimulation^35,36^, which may be more sensitively captured by SII. In contrast, the finding that both SII and NLR were significant in the group consuming 1–5 g of alcohol per day is difficult to interpret as a simple linear dose-response relationship. This pattern may have been influenced by differences in the number of cases across alcohol consumption categories, characteristics of the reference group, inclusion of former drinkers in the reference group, or measurement error inherent in self-reported alcohol intake. Therefore, the significant findings in the low alcohol consumption group are more appropriately interpreted as reflecting the complex and heterogeneous nature of alcohol-related patterns in this study.

### Strengths and limitations

This study has several strengths. First, the use of a large prospective cohort with approximately 14 years of follow-up allowed for a clearer assessment of temporal relationships. Second, stratified analyses were conducted not only in the overall male population but also among current smokers, current drinkers, and individuals with concurrent current smoking and drinking, enabling a comprehensive evaluation of the combined effects of systemic inflammatory markers and lifestyle factors. Third, multiple analytical approaches, including one standard deviation analysis, quartile analysis, trend analysis, joint exposure analysis, and analyses according to smoking and alcohol consumption levels, were employed to examine the robustness and patterns of the results. Fourth, the main findings were generally maintained even after additional adjustment for liver function markers and endogenous antioxidant biomarkers such as bilirubin, albumin, and uric acid, suggesting that the observed associations may not be explained solely by liver function or antioxidant status.

Several limitations should also be considered. First, SII and NLR were assessed using single baseline measurements, which do not account for temporal changes or variability over the follow-up period. Second, smoking and alcohol consumption were based on self-reported data, which may be subject to misclassification. In particular, alcohol intake may not have fully captured long-term exposure or complex drinking patterns. Third, women were excluded due to the very low prevalence of smoking, which limits the generalizability of the findings to women or the general population. Fourth, residual confounding cannot be completely excluded, and information on comorbidities, infectious conditions, or medication use that may influence systemic inflammation may have been limited. Fifth, the number of cases in certain subgroups, such as individuals with concurrent current smoking and drinking or specific categories of smoking and alcohol consumption, was relatively small, which may have resulted in uncertainty in the estimates.

## CONCLUSIONS

In this large prospective cohort study of Korean men, systemic inflammatory markers, particularly SII, were associated with increased lung cancer risk. These associations were more evident among individuals with current smoking, suggesting that systemic inflammatory status may reflect the biological burden associated with smoking-related carcinogenic processes. SII showed relatively consistent associations across multiple analytical approaches, indicating its potential utility as a marker for identifying individuals at higher risk of smoking-related lung cancer. Further studies are warranted to determine whether integrating systemic inflammatory markers with established behavioral risk factors can improve lung cancer risk stratification and prevention strategies.

## Data Availability

The data supporting this research are available from the authors on reasonable request. The datasets, including summary statistics and SAS code generated during the current study, are available. Due to privacy and ethical restrictions, raw data are not publicly available.
